# PET/CT radiomics for prediction of hyperprogression in metastatic melanoma patients treated with immune checkpoint inhibitors

**DOI:** 10.3389/fonc.2022.977822

**Published:** 2022-11-24

**Authors:** H. S. Gabryś, L. Basler, S. Burgermeister, S. Hogan, M. Ahmadsei, M. Pavic, M. Bogowicz, D. Vuong, S. Tanadini-Lang, R. Förster, K. Kudura, M. Huellner, R. Dummer, M. P. Levesque, M. Guckenberger

**Affiliations:** ^1^ Department of Radiation Oncology, University Hospital Zurich, University of Zurich, Zurich, Switzerland; ^2^ Department of Dermatology, University Hospital Zurich, University of Zurich, Zurich, Switzerland; ^3^ Department of Nuclear Medicine, University Hospital Zurich, University of Zurich, Zurich, Switzerland

**Keywords:** hyperprogression, melanoma, immune checkpoint inhibition, radiomics, PET/CT

## Abstract

**Purpose:**

This study evaluated pretreatment 2[18F]fluoro-2-deoxy-D-glucose (FDG)-PET/CT-based radiomic signatures for prediction of hyperprogression in metastatic melanoma patients treated with immune checkpoint inhibition (ICI).

**Material and method:**

Fifty-six consecutive metastatic melanoma patients treated with ICI and available imaging were included in the study and 330 metastatic lesions were individually, fully segmented on pre-treatment CT and FDG-PET imaging. Lesion hyperprogression (HPL) was defined as lesion progression according to RECIST 1.1 and doubling of tumor growth rate. Patient hyperprogression (PD-HPD) was defined as progressive disease (PD) according to RECIST 1.1 and presence of at least one HPL. Patient survival was evaluated with Kaplan-Meier curves. Mortality risk of PD-HPD status was assessed by estimation of hazard ratio (HR). Furthermore, we assessed with Fisher test and Mann-Whitney *U* test if demographic or treatment parameters were different between PD-HPD and the remaining patients. Pre-treatment PET/CT-based radiomic signatures were used to build models predicting HPL at three months after start of treatment. The models were internally validated with nested cross-validation. The performance metric was the area under receiver operating characteristic curve (AUC).

**Results:**

PD-HPD patients constituted 57.1% of all PD patients. PD-HPD was negatively related to patient overall survival with HR=8.52 (95%CI 3.47-20.94). Sixty-nine lesions (20.9%) were identified as progressing at 3 months. Twenty-nine of these lesions were classified as hyperprogressive, thereby showing a HPL rate of 8.8%. CT-based, PET-based, and PET/CT-based models predicting HPL at three months after the start of treatment achieved testing AUC of 0.703 +/- 0.054, 0.516 +/- 0.061, and 0.704 +/- 0.070, respectively. The best performing models relied mostly on CT-based histogram features.

**Conclusions:**

FDG-PET/CT-based radiomic signatures yield potential for pretreatment prediction of lesion hyperprogression, which may contribute to reducing the risk of delayed treatment adaptation in metastatic melanoma patients treated with ICI.

## Introduction

Immune checkpoint inhibition (ICI) targeting PD-1 and CTLA-4 have become guideline-recommended treatment standards in metastatic melanoma and have revolutionized the outcome of this disease (1–3). However, more than 50% of patients do not respond to treatment (4).

Moreover, one of the most important subjects is early identification and potentially prediction of patients who experience rapid disease progression, that is a hyperprogressive disease (PD-HPD), following ICI treatment (5–7). In this specific subset of patients, the treatment with ICI is actually harmful to patients and leads to a worsened outcome. About 4–29% of patients treated with ICI may experience PD-HPD (7). There is no widely accepted definition of PD-HPD, however it is often defined as progression (PD) in terms of RECIST criteria with at least doubling of the tumor growth rate (TGR) (6, 8, 9). The biological and clinical mechanisms leading to the development of PD-HPD are not yet fully understood.

Identifying biomarkers for ICI response prediction and response assessment is challenging. Currently, only LDH is established as a serum biomarker with prognostic value for overall survival (OS) in melanoma (10). S100 is another well-known blood marker, associated with response to Ipilimumab (11). However, despite obvious advantages of biospecimen-derived biomarkers, they require at least minimally invasive diagnostic techniques and are not used in the current clinical routine (12–20). On the other hand, non-invasive anatomical and functional imaging using CT, MRI, and FDG-PET is performed repetitively during the treatment course, providing the opportunity for continuous response evaluation. Furthermore, imaging-based biomarkers could be able to identify most likely response of every single lesion, whereas serum biomarkers can be correlated only with response on a patient level.

Quantitative medical image analysis with radiomics has been shown to predict not only the immune phenotype of tumors but also the clinical patient outcome (21). In our previous study, we presented FDG-PET/CT-based radiomic and delta-radiomic prediction models for the differentiation of pseudoprogression from true progressive disease (22). Recently, Wang et al. showed that CT-based radiomics has potential to predict early response to ICI and to identify pseudoprogression (23).

As of today, there are no reliable, clinically validated biomarkers of PD-HPD. In recent years, a few studies were published investigating suitability of image-based biomarkers for PD-HPD prediction (24–26). Most of these studies, however, considered only CT-based radiomic signatures. In this exploratory study, we aimed to evaluate the value of radiomic signatures for early prediction of PD-HPD from pre-treatment CT and FDG-PET imaging. Here, we investigate whether combination of CT-based and PET-based radiomics could provide an advantage over models based on a single imaging modality.

## Material and methods

### Patient cohort

This analysis is based on a retrospective cohort of 190 consecutive metastatic melanoma patients treated with either single checkpoint-inhibition (anti-PD-1) or dual checkpoint inhibition (anti-PD-1/anti-CTLA-4) between 2013 and 2019 in a single institution. The study was approved by the local ethics committee (Kantonale Ethikkommission Zürich, approval number 2019-01012) in accordance with ‘good clinical practice’ (GCP) guidelines and the Declaration of Helsinki. Written informed consent was obtained from all patients.

The following exclusion criteria were applied: lack of pre-baseline/baseline/follow-up imaging; patients with only contrast-enhanced CT imaging (as most patients were staged/followed with non-enhanced PET/CT-imaging); patients with only brain metastases; patients presenting with only very small metastases at baseline (all baseline lesions <0.5 cc). After exclusion of patients without valid baseline and follow-up imaging as well as at least one measurable non-brain metastasis at baseline, the cohort was reduced from 190 to 112 patients and corresponded to the cohort used in our previous study (22). Subsequently, 56 patients without pre-baseline imaging were excluded. This resulted in 56 patients that were selected for this study. The patient characteristics are provided in **Table 1**.

**Table 1:** Patient characteristics.

	All patients (n=56)	nPD (n=42)	PD-nHPD (n=6)	PD-HPD (n=8)	*p*-value
**Age (years)**					0.89
Median	70	70	67	66	
Q1-Q3	53–74	52–76	56–70	58–72	
Range	33–93	33-93	41–80	53–81	
**Sex**					0.67
Male	41 (73.2%)	32 (76.2%)	4 (66.7%)	5 (62.5%)	
Female	15 (26.8%)	10 (23.8%)	2 (33.3%)	3 (37.5%)	
Type of ICI*					1.00
aPD1	50 (89.3%)	38 (90.5%)	5 (83.3%)	7 (87.5%)	
aCTLA4 + aPD1	5 (8.9%)	4 (9.5%)	1 (16.7%)	0	
aCTLA4	1 (1.8%)	0	0	1 (12.5%)	
**Number of lesions**					0.19
Total	330	250	18	62	
Median	5	6	3	7	
Q1-Q3	2–8	2–9	1–4	5–10	
Range	1–19	1–19	1–6	2–15	
**Metastatic sites**					
Lymph node	120 (36.4%)	97 (38.8%)	9 (50.0%)	14 (22.6%)	0.42
Lung	61 (18.5%)	52 (20.8%)	3 (16.7%)	6 (9.7%)	0.62
Liver	38 (11.5%)	24 (9.6%)	1 (5.6%)	13 (21.0%)	0.06
Bone	32 (9.7%)	21 (8.4%)	0 (0.0%)	11 (17.7%)	0.75
Other	79 (23.9%)	56 (22.4%)	5 (27.8%)	18 (29.0%)	
BRAF mutation**					0.66
Wild type	42 (75.0%)	31 (73.8%)	04 (66.7%)	7 (87.5%)	
V600E	9 (16.1%)	6 (14.3%)	2 (33.3%)	1 (12.5%)	
V600K	2 (3.6%)	2 (4.8%)	0	0	
V600-K601E	1 (1.8%)	1 (2.4%)	0	0	
K601E	1 (1.8%)	1 (2.4%)	0	0	
N581S	1 (1.8%)	1 (2.4%)	0	0	
**New lesions during follow-up**					0.03
Yes	15 (26.8%)	5 (11.9%)	5 (83.3%)	5 (62.5%)	
No	41 (73.2%)	37 (88.1%)	1 (16.7%)	3 (37.5%)	
**LDH [U/L]**					0.27
Median	380	374	414	428	
Q1-Q3	343–462	326–441	382–463	362–500	
Range	247–1099	247–1099	299–759	337–639	
**S100 [μg/L]**					0.64
Median	0.140	0.145	0.105	0.200	
Q1-Q3	0.085–0.315	0.065–0.468	0.085–0.178	0.115–0.250	
Range	0.00–2.940	0.000–2.940	0.000–0.300	0.090–1.190	

* p-value for type of ICI was calculated as single vs double checkpoint inhibition.

** p-value for BRAF mutation was calculated as wild type vs (any) mutation. The *p*-values were reported for comparisons between PD-HPD vs. the remaining patients (nPD and PD-nHPD). nPD, non-progressing patients; PD-nHPD, progressing but not hyperprogressing patients; PD-HPD, hyperprogressin patients.

### Endpoints

The primary study endpoint was hyperprogression of individual metastatic lesions (HPL) at 3 months after the start of immunotherapy. The HPL was defined as progression according to RECIST criteria and at least doubling of a lesion’s TGR between the baseline and the follow-up (6, 8, 9). The TGR was estimated using the exponential tumor growth assumption following the approach of Champiat et al. (8). To establish the TGR, the long diameters (according to RECIST) of all lesions were measured at the pre-baseline (3 months +/- 2 months before the baseline), baseline (up to 3 months before the treatment), and follow-up (3 months +/- 1.5 months after the start of the treatment).

Additionally, we evaluated hyperprogression on a patient level, that is a hyperprogressing disease (PD-HPD). A patient with PD-HPD was defined as a patient developing a progressive disease (PD) according to the RECIST 1.1 criteria (27) who had at least one HPL. PD patients that were not hyperprogressing were marked as PD-nHPD, whereas patients that were not progressing were marked as nPD.

### Imaging and lesion delineation

All imaging was performed using standardized imaging-protocols at a single institution. The patients were injected with a body-weight-dependent and/or BMI-adapted FDG dose (2.0–3.5 MBq per kg). Scanning was performed on different scanners, partly with time-of-flight acquisition. PET image reconstructions used ordered subset expectation maximization together with point spread function modeling where available. The CT acquisition parameters were almost identical for all scanners and have been described previously in detail (28).

**Figure 1:**
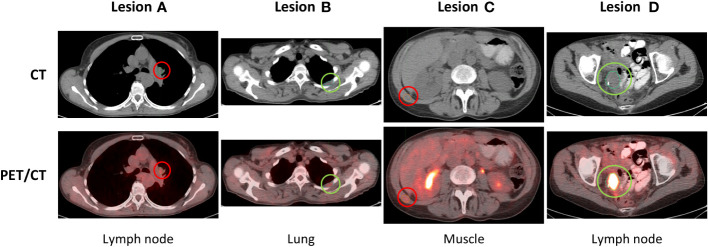
Baseline images of hyperprogressing **(A, C)** and non-hyperprogressing **(B, D)** lesions of a selected patient.

Fifty-six patients with a total of 330 lesions at baseline fulfilled all inclusion and exclusion criteria, which differed from our previous study in the regard that an additional pre-baseline imaging was required to define the pre-baseline TGR. All lesions were manually segmented in 3D and mutually checked by two experienced clinicians with validated reproducibility based on a common protocol and consistent quality control at all time-points. A rigid registration was performed for CT and PET images and CT-based contours were propagated to the PET images. Spatial mismatches were manually corrected by shifting the CT-based contours to the corresponding lesion locations in PET images. Subsequently, PET contours were adapted to conform to the PET signal. Sample baseline images with segmentations are presented in **Figure 1**.

### Radiomic feature extraction

Preprocessing and extraction of radiomic features from the baseline medical images were done using an in-house developed software, Z-Rad (29). Implementation of radiomic feature definitions in Z-Rad follows the Image Biomarker Standardization Initiative (IBSI) (30).

In the preprocessing step, CT and PET images were resized to isotropic voxels of 2 mm size. Intensity values of the images were discretized using a quantization step of 5 HU for CT and 0.25 SUV for PET. Lesions smaller than 0.5 cc were excluded from the analysis. Features describing shape of the lesions, histogram of voxel intensities, and texture of the lesions were extracted. Since CT-based and PET-based segmentations differed slightly, shape features were extracted separately from CT and PET images. In total, 180 radiomic features per lesion were extracted for each imaging modality. A full list of extracted features is provided in the supplement.

### Statistical analysis

The PD-HPD rates with respect to patient sex, the immune checkpoint inhibition type, BRAF mutation status, and appearance of new lesions at the follow-up were compared with Fisher’s exact test. Mann-Whitney *U* test was used to evaluate association of PD-HPD rate with patient age, total number of metastatic lesions, and serum biomarker levels. The difference in the HPL rate with respect to lesion location was evaluated with Fisher’s exact test.

Patient OS was estimated with the Kaplan-Meier estimator and the difference in survival between different patient groups was assessed using the log-rank test and hazard ratios. The landmark for the survival analysis was set at three months after start of treatment to correct for *guarantee-time bias* (31, 32). Therefore, all metrics relating to patient OS in this study are to be taken with respect to this landmark and not to the start of treatment.

The models of hyperprogression were trained to predict HPL at 3 months after start of the treatment based on baseline FDG-PET/CT imaging data. Building reliable models of PD-HPD was not feasible due to the low number of patients. Three HPL models were considered: 1) a model relying on CT-based radiomic features, 2) a model relying on PET-based radiomic features, 3) and a model relying on both CT- and PET-based radiomic features. The feature space comprised 180 radiomic features extracted from CT, 180 radiomic features extracted from PET images. Next, from all pairs of features correlated above Kendall’s *tau* = 0.90, a random one was subsequently removed to reduce redundancy of the features.

The predictive models of HPL were trained, optimized, and tested in a setting of a nested cross-validation. A nested cross-validation allows to select the best model for the data set and the best set of hyperparameters for the chosen model. Hyperparameter selection (model tuning) is done in the inner cross-validation, whereas the outer cross-validation estimates an unbiased performance of the model (33). The inner loop, used for model tuning, was a 10x-repeated stratified 5-fold cross-validation. In total, 100 randomly generated hyperparameter samples were evaluated in model tuning with random search optimization. The list and scope of hyperparameters that were tuned is provided in the code repository associated with this manuscript. The outer loop, used for model testing, was a stratified group 5-fold cross-validation. This type of cross-validation ensured that folds preserved the percentage of samples for each class and lesions of the same patients were not overlapping testing and training folds. Feature selection method and its hyperparameters were part of the model tuning pipeline. It was realized by fitting a gradient tree boosting model and selection of its most important features. These features were later fed into a logistic regression model (34). We imposed an arbitrary hard limit of maximum six features per imaging modality to avoid generation of overly complex models. For this reason, the largest CT-based and PET-based models had six covariates, whereas the largest PET/CT-based models had twelve covariates.

Model performance was measured with the area under receiver operating characteristic curve (AUC). The variability of the performance scores was estimated with standard deviation of the scores from the cross-validation.

The following open-source Python packages were used for visualization and statistical analysis: Lifelines (35), Matplotlib (36), NumPy & SciPy (37), Pandas (38), Scikit-learn (39), XGBoost (40). The code used for model training, tuning, testing, and visualization is provided in a public online repository: https://github.com/hubertgabrys/PET-CT-radiomics-for-prediction-of-hyperprogression-in-metastatic-melanoma-patients-treated-with-imm

## Results

### Hyperprogression rates and patient survival

**Figure 2:**
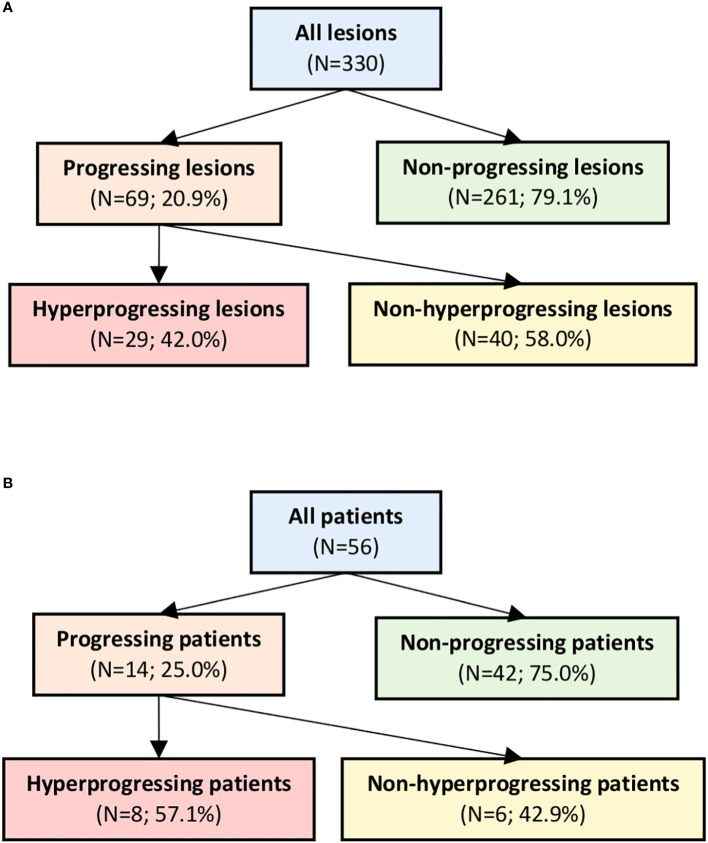
Progression and hyperprogression rates on a lesion **(A)** and a patient level **(B)**.

The cohort comprised 56 patients with 330 lesions at baseline (**Figure 2**). According to the RECIST criteria, 20.9% (n=69) lesions were progressing. The 29 hyperprogressing lesions corresponded to a 42.0% HPL rate among the progressing lesions and an 8.8% HPL rate among all lesions. The difference in HPL rates with respect to lesion location was evaluated with Fisher’s exact test and did not result in significant differences: lymph nodes (*p*-value=0.42), lung (*p*-value=0.62), liver (*p*-value=0.06), and bone (*p*-value=0.75).

The 14 PD patients accounted for 25.0% of all patients. The PD-HPD patients (n=8) constituted 57.1% of PD patients and 14.3% of all patients. The influence of various patient-specific factors on the PD-HPD rate was evaluated and the results are reported in **Table 1**. PD-HPD was independent from patient age (*p*-value=0.89) and sex (*p*-value=0.67). Also, BRAF mutation status (*p*-value=0.66) and the type of immune checkpoint inhibition (*p*-value=1.00) were not significantly different between the groups PD-HPD and the remaining patients. A slight trend of increasing median values of LDH from nPD through PD-nHPD to PD-HPD was observed, however the difference was far from statistical significance (*p*-value=0.27). The other analyzed serum biomarker, that is S100, also was not significant (*p*-value=0.64). Total number of lesions at baseline was not associated with risk of PD=HPD (*p*-value=0.19). However, appearance of new metastatic lesions during follow-up was significantly higher in the PD-HPD group compared to the other patients with *p*-value=0.03.

**Figure 3:**
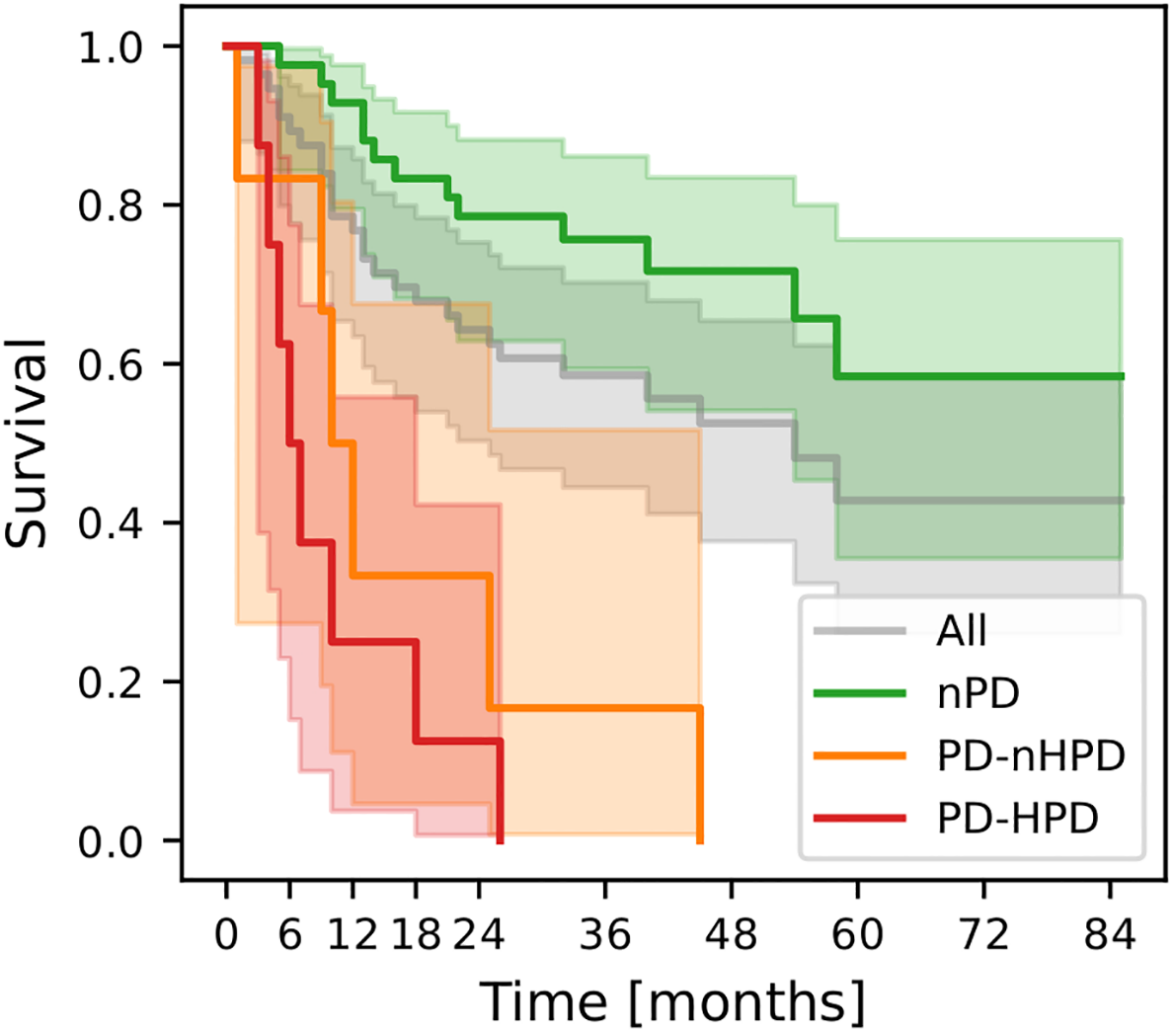
Kaplan-Meier curves for the whole cohort (All; n=56), non-progressing patients (nPD; n=42), progressing but not hyperprogressing patients (PD-nHPD; n=6), and hyperprogressing patients (PD-HPD; n=8). Survival functions were estimated with respect to the landmark (3 months) and not to the start of treatment.

Median survival in the total cohort was 54 months. The nPD patients had significantly longer survival (median OS not reached; HR = 0.10 (95%CI 0.05-0.23)) than PD-nHPD (median OS = 12 months; HR = 4.27 (95%CI 1.69-10.81)) and PD-HPD (median OS = 7 months; HR = 8.52 (95%CI 3.47-20.94)) patients (*p*-value<0.01). However, the difference in survival between PD-nHPD and PD-HPD was not statistically significant (*p*-value = 0.32). Kaplan-Meier curves for different patient groups are presented in **Figure 3**.

### Models of hyperprogression

After removal of correlations above Kendall’s *tau* = 0.90, the dimensionality was reduced to 85 CT-based features and 78 PET-based features. Full correlation matrix is provided in the supplement. The dendrogram showing hierarchical agglomerative clustering of features left after the dimensionality reduction is also provided in the supplement.

**Figure 4:**
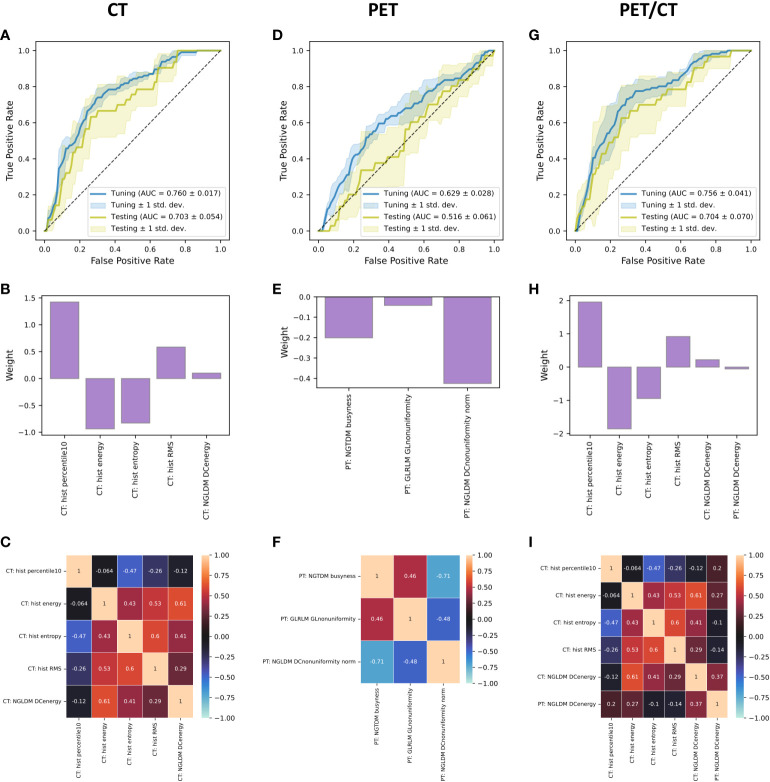
Receiver operating characteristic curves **(A, D, G)**, feature weights **(B, E, H)**, and correlation matrices with Kendall’s *tau* **(C, F, I)** for the CT-, PET-, and PET/CT-based logistic regression models predicting HPL at three months after start of treatment. The receiver operating characteristic curves were calculated for each fold of cross-validation and the curves presented in the figure are result of averaging of the component curves. Python code used for generation of these figures is provided in a public online repository (https://github.com/hubertgabrys/PET-CT-radiomics-for-prediction-of-hyperprogression-in-metastatic-melanoma-patients-treated-with-imm).

The CT-based model achieved AUC=0.760 +/- 0.017 in training and AUC=0.703 +/- 0.054 in testing (**Figure 4A**). The model was based on four histogram features and one texture feature (**Figure 4B**). However, the weight of the texture feature was rather low and had little influence on model predictions. There were we no highly correlated features in the model (**Figure 4C**). The PET-based model failed to validate achieving AUC=0.629 +/- 0.028 in training and AUC=0.516 +/- 0.061 in testing (**Figure 4D**). The model weights and correlations among model covariates are presented in **Figures 4E, F**). The PET/CT model performed comparable to the CT-based model scoring AUC=0.756 +/- 0.041 in training and AUC=0.704 +/- 0.070 in testing (**Figure 4G**). The model was based on the same five features as the CT-based model with the addition of one PET-based texture feature (**Figure 4H**). Both CT-based and PET-based texture features in this model had relatively low weights so the model outputs were mainly driven by the CT-based histogram features. A correlation matrix of the model covariates is presented in **Figure 4I**.

## Discussion

Hyperprogression, which describes an accelerated disease progression, constitutes a major challenge in treatment of cancer patients treated with ICI. In this study, we investigated the prevalence of PD and PD-HPD in our patient cohort and its relation to patient OS. Furthermore, we trained and tested machine learning models based on pre-treatment FDG-PET/CT radiomics to predict and identify metastatic lesions, which might become HPL at 3 months after start of therapy.

The incidence of PD-HPD described in previous studies was between 4-29% across multiple histologies (7). Consistently with the existing literature, the PD-HPD rate in our study was 8.8% on a metastatic lesion level distributed over 8 patients. It is noteworthy that the PD-HPD patients constituted 57.1% of all PD patients. This shows that, although PD-HPD affects a relatively small fraction of all patients, it appears to form a significant proportion within a group of patients that are in a progressing disease stage. Furthermore, PD-HPD is a high-risk factor and was associated with significantly reduced OS compared to the other patients (median OS = 7 months vs. “not reached”), regardless of the type of ICI. Several other studies have reported similar results (7, 24). An explanation might be that the affected patients experienced a negative effect upon treatment with ICI (41). While the exact pathomechanism of PD-HPD is not clearly understood yet, several studies suggest that a variety of immune-cells might play a crucial role. Upon treatment with ICI, PD-HPD patients show a massive increase of intratumoral macrophages, which can express PD1 and thereby block ICI. Furthermore, PD-HPD patients show an increased amount of senescent CD4+ T cells (Tsens) and Ki67-positive effector regulatory T cells in tumor-infiltrating lymphocytes (TIL) (42). We also observed a significantly increased rate of new metastases in PD-HPD patients during the first three months of the follow-up (*p*-value = 0.03). The difference in the OS between PD-HPD and PD-nHPD patients was noticeable (median OS = 7 months and median OS = 12 months, respectively), however statistically not significant, likely due to a limited number of patients in these groups.

The potential of radiomics to predict treatment response and outcome for a variety of tumor types has been demonstrated in several studies (43, 44). In our previous study, we demonstrated that the combination of multi-modal radiomics and blood markers can differentiate true progression from pseudoprogression in melanoma patients treated with ICI (22). Vaidya et al. reported that CT-based radiomic signatures extracted from pre-treatment CTs of patients with advanced NSCLC treated with ICI can identify patients at risk of hyperprogression (AUC=0.96) (25). Unfortunately, no uncertainty estimates were provided. Additionally, Song et al. evaluated CT-based radiomic features of hyperprogression at the lesion level in patients with advanced lung cancer treated with ICI (24). The authors demonstrated that radiomic features identifying hyperprogression differed among organs with AUC varying from 0.61 to 0.72, highlighting the potential of radiomics to differentiate hyperprogression and its organ-specific micro-environment. In both studies, HPD correlated with significantly reduced OS. Predictive performance of our models (AUC=0.70) is in line with the scores reported by Song et al. Furthermore, He et al. showed in a multicenter study that pre-treatment CT-based radiomics might contribute to predict atypical responses to ICI in several cancer types (26). The models reported by He et al. achieved higher AUCs ranging from 0.77 to 0.93.

CT-based radiomic features proved to be more predictive of HPL than PET-based features. Our CT-based model achieved generalization AUC=0.703 +/- 0.054. The PET-based model was meaningless (AUC testing=0.516 +/- 0.061). Moreover, the PET/CT did not provide significant improvement over the CT-based model (AUC testing=0.704 +/- 0.070) and virtually resembled the CT-based model with addition of one texture type PET-based feature. This indicates that PET imaging does not provide additional hyperprogression-related information over CT in our data set. This could be caused by relatively low imaging resolution of PET with respect to lesion size.

The CT-based features underlying the models were mostly intensity features. Interpretation of results in studies involving radiomics are always challenging. Nevertheless, the features underlying our best performing models paint a coherent picture. It seems that lesions classified as likely to become hyperprogressive had high minimum intensity values (high hist_percentile10) and high average signal intensity (high hist_RMS). However, at the same time these lesions had relatively low overall signal intensity (low hist energy) and their intensity distribution was rather homogenous (low hist_entropy). This most likely means that a typical, according to our model, lesion that is at risk of hyperprogression has elevated uniform average intensity with no hot- or cold spots.

The presented CT-based and PET/CT-based models could not serve the purpose of clinical tools identifying with high certainty which lesion is going to become hyperprogressive in the future. However, they identify lesions that are at higher risk of becoming hyperprogressive. Identification of such lesions already at baseline could allow for closer patient monitoring or early treatment adaptations. Furthermore, lack of reliance on PET-based radiomics could reduce cost and time of image acquisition for these models.

There are a few limitations of this study. Those include the retrospective nature of the analysis and a limited total number of patients. The segmentation of metastatic lesions was done manually which inherently involves a degree of inter- and intraobserver variability. To reduce it, we performed qualitative segmentation stability control by ensuring that both observers followed the same protocol and segmentations were cross-checked and corrected when necessary. Moreover, no external validation of our models has been performed, yet. In order to circumvent this challenge, we did an internal validation with nested cross-validation which is a robust unbiased method of generalization performance estimation. Nevertheless, despite these limitations, this study is the largest multimodal FDG-PET/CT radiomics analysis conducted to predict hyperprogression in metastatic melanoma patients treated with ICI.

## Conclusion

This study analyzed multimodal radiomic signatures of hyperprogression in metastatic melanoma patients treated with ICI. Our CT- and PET/CT-based models were able to predict development of HPL at 3 months after start of the treatment based on the baseline imaging data. Therefore, we showed that performing PET/CT radiomics at baseline can help to early recognize lesions that are likely to become HPL and potentially patients at risk of PD-HPD. This could allow for early treatment adaptation, increased patient monitoring to improve treatment outcome, and reduction of costs related to unnecessary ICI therapy.

## Data availability statement

The raw data supporting the conclusions of this article will be made available by the authors, without undue reservation.

## Ethics statement

The studies involving human participants were reviewed and approved by the local ethics committee (Kantonale Ethikkommission Zürich, approval number 2019-01012) in accordance with ‘good clinical practice’ (GCP) guidelines and the Declaration of Helsinki. Written informed consent was obtained from all patients. The patients/participants provided their written informed consent to participate in this study.

## Author contributions

Concept and design: ML, RD, MG, LB, RF, MB, ST-L. Acquisition, analysis, or interpretation of data: HG, LB, SB. Statistical analysis: HG. Obtained funding: LB, HG, SH, ML, MG. Study supervision: ML, MG. Writing, review, and revision of the manuscript: All authors. All authors contributed to the article and approved the submitted version.

## Funding

CRC (Cancer Research Center) Funding program (CRC_13), Comprehensive Cancer Center Zurich, University Hospital Zurich, Zurich, Switzerland; Swiss National Fund (SNF 310030_170159); European Training Network MELGEN funded consortium No. 641458; Maiwand Ahmadsei received support through the “Young Talents in Clinical Research” Beginner’s Grant from the Swiss Academy of Medical Sciences (SAMW) and the Bangerter-Rhyner Foundation.

## Conflict of interest

KK: Grant from the Iten-Kohaut-Foundation. MH: Speaker’s fees and grants from GE Healthcare. Grant for translational and clinical cardiac and oncological research from the Alfred and Annemarie von Sick legacy. Grant from the Artificial Intelligence in Oncological Imaging Network by the University of Zurich. RD: Intermittent, project focused consulting and/or advisory relationships with Novartis, Merck Sharp & Dhome MSD, Bristol-Myers Squibb BMS, Roche, Amgen, Takeda, Pierre Fabre, Sun Pharma, Sanofi, Catalym, Second Genome, Regeneron, Alligator, T3 Pharma, MaxiVAX SA, Pfizer and touchIME. Funding: URPP and Skintegrity. ML: Intermittent, project specific research funding from Roche, Novartis, Molecular Partners, and Oncobit AG.

The remaining authors declare that the research was conducted in the absence of any commercial or financial relationships that could be construed as a potential conflict of interest.

## Publisher’s note

All claims expressed in this article are solely those of the authors and do not necessarily represent those of their affiliated organizations, or those of the publisher, the editors and the reviewers. Any product that may be evaluated in this article, or claim that may be made by its manufacturer, is not guaranteed or endorsed by the publisher.
