# Supplementary material for: PET/CT radiomics for prediction of hyperprogression in metastatic melanoma patients treated with immune checkpoint inhibitors
Source: Front Oncol. 2022 Nov 24;12:977822. doi: 10.3389/fonc.2022.977822 (PMC9730880; doi:10.3389/fonc.2022.977822)
Supplement: Supplementary file 1 [file Table_1.pdf]

# Extracted features

| Index | Standardization Name | Type | Matrix | Merging |
|---|---|---|---|---|
| 1 | volume | Shape | NA | NA |
| 2 | surface | Shape | NA | NA |
| 3 | compactness_1 | Shape | NA | NA |
| 4 | compactness_2 | Shape | NA | NA |
| 5 | spherical disproportion | Shape | NA | NA |
| 6 | sphericity | Shape | NA | NA |
| 7 | asphericity | Shape | NA | NA |
| 8 | surface to volume ratio | Shape | NA | NA |
| 9 | median thickness | Shape | NA | NA |
| 10 | standard deviation thickness | Shape | NA | NA |
| 11 | euclidian distance | Shape | NA | NA |
| 12 | major axis length | Shape | NA | NA |
| 13 | minor axis length | Shape | NA | NA |
| 14 | least axis length | Shape | NA | NA |
| 15 | elongation | Shape | NA | NA |
| 16 | flatness | Shape | NA | NA |
| 17 | fractal dimension | Shape | NA | NA |
| 18 | center of mass shift | Shape | NA | NA |
| 19 | MaxIntensityTumorVolume20% | Histogram | NA | NA |
| 20 | MaxIntensityTumorVolume30% | Histogram | NA | NA |
| 21 | MaxIntensityTumorVolume40% | Histogram | NA | NA |
| 22 | MaxIntensityTumorVolume50% | Histogram | NA | NA |
| 23 | MaxIntensityTumorVolume60% | Histogram | NA | NA |
| 24 | MaxIntensityTumorVolume70% | Histogram | NA | NA |
| 25 | minimum | Histogram | NA | NA |
| 26 | maximum | Histogram | NA | NA |
| 27 | mean | Histogram | NA | NA |
| 28 | standard deviation | Histogram | NA | NA |

| | | | | |
|---|---|---|---|---|
| 29 | coefficient of variation | Histogram | NA | NA |
| 30 | skewness | Histogram | NA | NA |
| 31 | kurtosis | Histogram | NA | NA |
| 32 | variance | Histogram | NA | NA |
| 33 | median | Histogram | NA | NA |
| 34 | percentile 10th | Histogram | NA | NA |
| 35 | percentile 90th | Histogram | NA | NA |
| 36 | interquartile range | Histogram | NA | NA |
| 37 | range | Histogram | NA | NA |
| 38 | mean absolut deviation | Histogram | NA | NA |
| 39 | robust mean absolut deviation | Histogram | NA | NA |
| 40 | energy | Histogram | NA | NA |
| 41 | entropy | Histogram | NA | NA |
| 42 | root mean square | Histogram | NA | NA |
| 43 | uniformity | Histogram | NA | NA |
| 44 | energy | Texture | GLCM | No |
| 45 | entropy | Texture | GLCM | No |
| 46 | contrast | Texture | GLCM | No |
| 47 | correlation | Texture | GLCM | No |
| 48 | homogeneity | Texture | GLCM | No |
| 49 | homogeneity normalized | Texture | GLCM | No |
| 50 | inverese difference | Texture | GLCM | No |
| 51 | inverese difference normalized | Texture | GLCM | No |
| 52 | variance | Texture | GLCM | No |
| 53 | sum of average | Texture | GLCM | No |
| 54 | sum of entropy | Texture | GLCM | No |
| 55 | sum of variance | Texture | GLCM | No |
| 56 | difference entropy | Texture | GLCM | No |
| 57 | difference variance | Texture | GLCM | No |
| 58 | information measures of correlation 1 | Texture | GLCM | No |
| 59 | information measures of correlation 2 | Texture | GLCM | No |
| 60 | maximal correlation coefficient | Texture | GLCM | No |

| | | | | |
|---|---|---|---|---|
| 61 | joint maximum | Texture | GLCM | No |
| 62 | joint average | Texture | GLCM | No |
| 63 | difference average | Texture | GLCM | No |
| 64 | dissimilarity | Texture | GLCM | No |
| 65 | inverse variance | Texture | GLCM | No |
| 66 | autocorrelation | Texture | GLCM | No |
| 67 | cluster tendency | Texture | GLCM | No |
| 68 | cluster shade | Texture | GLCM | No |
| 69 | cluster prominence | Texture | GLCM | No |
| 70 | energy | Texture | mGLCM | Yes |
| 71 | entropy | Texture | mGLCM | Yes |
| 72 | contrast | Texture | mGLCM | Yes |
| 73 | correlation | Texture | mGLCM | Yes |
| 74 | homogeneity | Texture | mGLCM | Yes |
| 75 | homogeneity normalized | Texture | mGLCM | Yes |
| 76 | inverese difference | Texture | mGLCM | Yes |
| 77 | inverese difference normalized | Texture | mGLCM | Yes |
| 78 | variance | Texture | mGLCM | Yes |
| 79 | sum of average | Texture | mGLCM | Yes |
| 80 | sum of entropy | Texture | mGLCM | Yes |
| 81 | sum of variance | Texture | mGLCM | Yes |
| 82 | difference entropy | Texture | mGLCM | Yes |
| 83 | difference variance | Texture | mGLCM | Yes |
| 84 | information measures of correlation 1 | Texture | mGLCM | Yes |
| 85 | information measures of correlation 2 | Texture | mGLCM | Yes |
| 86 | maximal correlation coefficient | Texture | mGLCM | Yes |
| 87 | joint maximum | Texture | mGLCM | Yes |
| 88 | joint average | Texture | mGLCM | Yes |
| 89 | difference average | Texture | mGLCM | Yes |
| 90 | dissimilarity | Texture | mGLCM | Yes |
| 91 | inverse variance | Texture | mGLCM | Yes |
| 92 | autocorrelation | Texture | mGLCM | Yes |

| | | | | |
|---|---|---|---|---|
| 93 | cluster tendency | Texture | mGLCM | Yes |
| 94 | cluster shade | Texture | mGLCM | Yes |
| 95 | cluster prominence | Texture | mGLCM | Yes |
| 96 | coarseness | Texture | NGTDM | No |
| 97 | contrast | Texture | NGTDM | No |
| 98 | busyness | Texture | NGTDM | No |
| 99 | complexity | Texture | NGTDM | No |
| 100 | strength | Texture | NGTDM | No |
| 101 | grey level non-uniformity | Texture | GLRLM | No |
| 102 | grey level non-uniformity normalized | Texture | GLRLM | No |
| 103 | zone size non-uniformity | Texture | GLRLM | No |
| 104 | zone size non-uniformity normalized | Texture | GLRLM | No |
| 105 | short runs emphasis | Texture | GLRLM | No |
| 106 | long runs emphasis | Texture | GLRLM | No |
| 107 | low grey level run emphasis | Texture | GLRLM | No |
| 108 | high grey level run emphasis | Texture | GLRLM | No |
| 109 | short run low grey level emphasis | Texture | GLRLM | No |
| 110 | short run high grey level emphasis | Texture | GLRLM | No |
| 111 | long run low grey level emphasis | Texture | GLRLM | No |
| 112 | long run high grey level emphasis | Texture | GLRLM | No |
| 113 | run percentage | Texture | GLRLM | No |
| 114 | grey level variance | Texture | GLRLM | No |
| 115 | run length variance | Texture | GLRLM | No |
| 116 | run entropy | Texture | GLRLM | No |
| 117 | grey level non-uniformity | Texture | mGLRLM | Yes |
| 118 | grey level non-uniformity normalized | Texture | mGLRLM | Yes |
| 119 | zone size non-uniformity | Texture | mGLRLM | Yes |
| 120 | zone size non-uniformity normalized | Texture | mGLRLM | Yes |
| 121 | short runs emphasis | Texture | mGLRLM | Yes |
| 122 | long runs emphasis | Texture | mGLRLM | Yes |
| 123 | low grey level run emphasis | Texture | mGLRLM | Yes |
| 124 | high grey level run emphasis | Texture | mGLRLM | Yes |

| | | | | |
|---|---|---|---|---|
| 125 | short run low grey level emphasis | Texture | mGLRLM | Yes |
| 126 | short run high grey level emphasis | Texture | mGLRLM | Yes |
| 127 | long run low grey level emphasis | Texture | mGLRLM | Yes |
| 128 | long run high grey level emphasis | Texture | mGLRLM | Yes |
| 129 | run percentage | Texture | mGLRLM | Yes |
| 130 | grey level variance | Texture | mGLRLM | Yes |
| 131 | run length variance | Texture | mGLRLM | Yes |
| 132 | run entropy | Texture | mGLRLM | Yes |
| 133 | grey level non-uniformity | Texture | GLSZM | No |
| 134 | grey level non-uniformity normalized | Texture | GLSZM | No |
| 135 | zone size non-uniformity | Texture | GLSZM | No |
| 136 | zone size non-uniformity normalized | Texture | GLSZM | No |
| 137 | small zone emphasis | Texture | GLSZM | No |
| 138 | large zone emphasis | Texture | GLSZM | No |
| 139 | low grey level zone emphasis | Texture | GLSZM | No |
| 140 | high grey level zone emphasis | Texture | GLSZM | No |
| 141 | small zone low grey level emphasis | Texture | GLSZM | No |
| 142 | small zone high grey level emphasis | Texture | GLSZM | No |
| 143 | large zone low grey level emphasis | Texture | GLSZM | No |
| 144 | large zone high grey level emphasis | Texture | GLSZM | No |
| 145 | zone percentage | Texture | GLSZM | No |
| 146 | grey level variance | Texture | GLSZM | No |
| 147 | zone size variance | Texture | GLSZM | No |
| 148 | zone size entropy | Texture | GLSZM | No |
| 149 | grey level non-uniformity | Texture | GLDZM | No |
| 150 | grey level non-uniformity normalized | Texture | GLDZM | No |
| 151 | zone size non-uniformity | Texture | GLDZM | No |
| 152 | zone size non-uniformity normalized | Texture | GLDZM | No |
| 153 | small distance emphasis | Texture | GLDZM | No |
| 154 | large distance emphasis | Texture | GLDZM | No |
| 155 | low grey level zone emphasis | Texture | GLDZM | No |
| 156 | high grey level zone emphasis | Texture | GLDZM | No |

| | | | | |
|---|---|---|---|---|
| 157 | small distance low grey level emphasis | Texture | GLDZM | No |
| 158 | small distance high grey level emphasis | Texture | GLDZM | No |
| 159 | large distance low grey level emphasis | Texture | GLDZM | No |
| 160 | large distance high grey level emphasis | Texture | GLDZM | No |
| 161 | zone percentage | Texture | GLDZM | No |
| 162 | grey level variance | Texture | GLDZM | No |
| 163 | zone distance variance | Texture | GLDZM | No |
| 164 | zone distance entropy | Texture | GLDZM | No |
| 165 | grey level non-uniformity | Texture | NGLDM | No |
| 166 | grey level non-uniformity normalized | Texture | NGLDM | No |
| 167 | dependence count non-uniformity | Texture | NGLDM | No |
| 168 | dependence count non-uniformity normalized | Texture | NGLDM | No |
| 169 | low dependence emphasis | Texture | NGLDM | No |
| 170 | high dependence emphasis | Texture | NGLDM | No |
| 171 | low grey level count emphasis | Texture | NGLDM | No |
| 172 | high grey level count emphasis | Texture | NGLDM | No |
| 173 | low dependence low grey level emphasis | Texture | NGLDM | No |
| 174 | low dependence high grey level emphasis | Texture | NGLDM | No |
| 175 | high dependence low grey level emphasis | Texture | NGLDM | No |
| 176 | high dependence high grey level emphasis | Texture | NGLDM | No |
| 177 | grey level variance | Texture | NGLDM | No |
| 178 | dependence count variance | Texture | NGLDM | No |
| 179 | dependence count entropy | Texture | NGLDM | No |
| 180 | dependence count energy | Texture | NGLDM | No |
