# Supplementary figures and images for: PET/CT radiomics for prediction of hyperprogression in metastatic melanoma patients treated with immune checkpoint inhibitors
Source: Front Oncol. 2022 Nov 24;12:977822. doi: 10.3389/fonc.2022.977822 (PMC9730880; doi:10.3389/fonc.2022.977822)

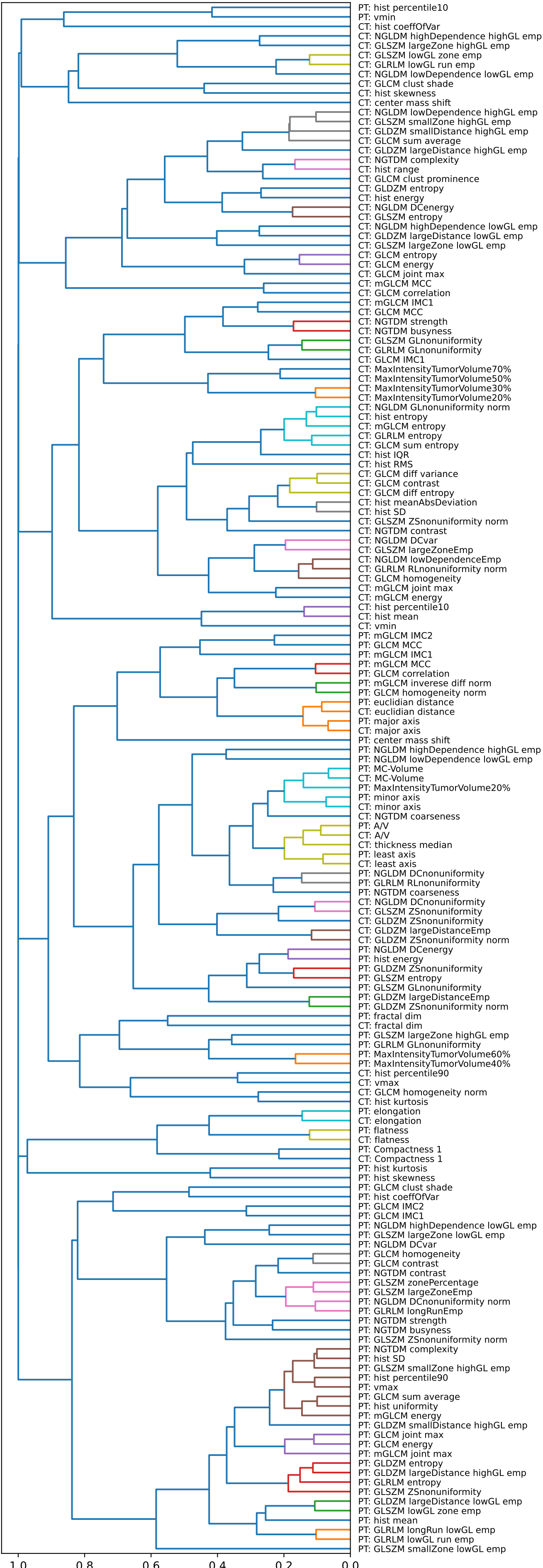

Supplement: Supplementary file 3 [file Image_1.pdf]
